# Supplementary material for: Diagnostic activity impacts lifetime risk of prostate cancer diagnosis more strongly than life expectancy
Source: PLoS One. 2022 Nov 23;17(11):e0277784. doi: 10.1371/journal.pone.0277784 (PMC9683621; doi:10.1371/journal.pone.0277784)
Supplement: S1 Table — (DOCX) [file pone.0277784.s001.docx]

**S1 table.** Lifetime risk of prostate cancer overall and per risk category according to intensity of diagnostic activity

|  | **Low (Sweden 1992)** | | | | | | **Intermediate (Sweden 2016)** | | | | | **High (Stockholm 2014*)** | | | | | |
| --- | --- | --- | --- | --- | --- | --- | --- | --- | --- | --- | --- | --- | --- | --- | --- | --- | --- |
| **Risk category** | **80 y** | **95% CI** | **100 y** | | | **95% CI** | **80 y** | **95% CI** | **100 y** | | **95% CI** | **80 y** | | **95% CI** | **100 y** | | **95% CI** |
| All | 10.5 | (9.7-11.3) | | 17.8 | (16.9-18.8) | | 16.6 | (15.7-17.6) | | 21.4 | (20.4-22.3) | | 24.8 | (21.4-28.1) | | 28.6 | (25.1-32.2) |
| Low-risk | 1.3 | (1.1-1.5) | | 1.8 | (1.5-2) | | 4.5 | (4.1-4.9) | | 4.8 | (4.4-5.2) | | 10.4 | (7.4-13.4) | | 10.7 | (7.6-13.7) |
| Intermediate-risk | 1.9 | (1.6-2.2) | | 2.8 | (2.4-3.2) | | 6.7 | (6-7.4) | | 7.5 | (6.8-8.2) | | 10.9 | (7.6-14.2) | | 11.6 | (8.2-15.1) |
| High-risk | 3.4 | (3-3.8) | | 5.7 | (5.2-6.2) | | 3 | (2.6-3.4) | | 4.4 | (4-4.8) | | 1.9 | (1.2-2.7) | | 2.9 | (2-3.8) |
| Regional metastases | 1.5 | (1.3-1.8) | | 2.8 | (2.5-3.1) | | 1 | (0.8-1.1) | | 1.7 | (1.5-1.9) | | 0.8 | (0.4-1.2) | | 1.4 | (0.9-1.9) |
| Distant metastases | 2.4 | (2.2-2.6) | | 4.8 | (4.4-5.1) | | 1.4 | (1.3-1.6) | | 3 | (2.7-3.3) | | 0.8 | (0.4-1.3) | | 2.1 | (1.4-2.7) |
| Low or intermediate-risk | 3.2 | (2.8-3.6) | | 4.5 | (4-5.1) | | 11.2 | (10.4-12.1) | | 12.3 | (11.4-13.2) | | 21.3 | (17.8-24.7) | | 22.3 | (18.8-25.9) |
| High-risk or metastatic | 7.3 | (6.7-7.9) | | 13.3 | (12.6-14) | | 5.4 | (5-5.9) | | 9.1 | (8.4-9.7) | | 3.5 | (2.5-4.6) | | 6.3 | (4.9-7.8) |

Life expectancy is set to correspond to men born 1992.

*A year when the Stockholm-3 study invited men to measure their PSA^15^.
