# Supplementary material for: Diagnostic activity impacts lifetime risk of prostate cancer diagnosis more strongly than life expectancy
Source: PLoS One. 2022 Nov 23;17(11):e0277784. doi: 10.1371/journal.pone.0277784 (PMC9683621; doi:10.1371/journal.pone.0277784)
Supplement: S2 Table — (DOCX) [file pone.0277784.s002.docx]

**S2 table.** Lifetime risk of prostate cancer overall and per category according to life expectancy for three birth cohorts.

|  | **Short (Birth year 1912)** | | | | **Intermediate (Birth year 1952)** | | | | **Long (Birth year 1992)** | | | |
| --- | --- | --- | --- | --- | --- | --- | --- | --- | --- | --- | --- | --- |
| **Risk category** | **80 y** | **95% CI** | **100 y** | **95% CI** | **80 y** | **95% CI** | **100 y** | **95% CI** | **80 y** | **95% CI** | **100 y** | **95% CI** |
| All | 13.2 | (12.4-14) | 14.7 | (13.9-15.5) | 15.3 | (14.5-16.2) | 18.4 | (17.6-19.3) | 16.6 | (15.7-17.6) | 21.4 | (20.4-22.3) |
| Low-risk | 3.7 | (3.4-4.1) | 3.8 | (3.5-4.2) | 4.2 | (3.8-4.6) | 4.4 | (4-4.8) | 4.5 | (4.1-4.9) | 4.8 | (4.4-5.2) |
| Intermediate-risk | 5.4 | (4.8-6) | 5.7 | (5.1-6.3) | 6.2 | (5.6-6.9) | 6.8 | (6.1-7.4) | 6.7 | (6-7.4) | 7.5 | (6.8-8.2) |
| High-risk | 2.3 | (2-2.6) | 2.7 | (2.4-3) | 2.7 | (2.4-3) | 3.7 | (3.3-4) | 3 | (2.6-3.4) | 4.4 | (4-4.8) |
| Regional metastases | 0.7 | (0.6-0.9) | 1 | (0.8-1.1) | 0.9 | (0.7-1) | 1.4 | (1.2-1.5) | 1 | (0.8-1.1) | 1.7 | (1.5-1.9) |
| Distant metastases | 1.1 | (0.9-1.2) | 1.5 | (1.4-1.7) | 1.3 | (1.1-1.4) | 2.3 | (2.1-2.5) | 1.4 | (1.3-1.6) | 3 | (2.7-3.3) |
| Low or intermediate-risk | 9.1 | (8.4-9.9) | 9.5 | (8.8-10.3) | 10.4 | (9.6-11.3) | 11.2 | (10.3-12) | 11.2 | (10.4-12.1) | 12.3 | (11.4-13.2) |
| High-risk or metastatic | 4.1 | (3.7-4.4) | 5.2 | (4.8-5.6) | 4.9 | (4.5-5.3) | 7.3 | (6.7-7.8) | 5.4 | (5-5.9) | 9.1 | (8.4-9.7) |

Diagnostic activity corresponding to ‘intermediately high’ as in Sweden in 2016.
